# Difference in Quality of Vision Outcome among Extended Depth of Focus, Bifocal, and Monofocal Intraocular Lens Implantation

**DOI:** 10.3390/healthcare10061000

**Published:** 2022-05-28

**Authors:** Chen-Cheng Chao, Hung-Yuan Lin, Chia-Yi Lee, Elsa Lin-Chin Mai, Ie-Bin Lian, Chao-Kai Chang

**Affiliations:** 1 Nobel Eye Institute, Taipei 100008, Taiwan; aachengachen@gmail.com (C.-C.C.); ao6u.3msn@hotmail.com (C.-Y.L.); 2 Department of Optometry, MacKay Junior College of Medicine, Nursing, and Management, Taipei 11260, Taiwan; elsamark@yahoo.com; 3 Universal Eye Center, Zhongli 32044, Taiwan; hylinmd@ms14.hinet.net; 4 Department of Optometry, Yuanpei University of Medical Technology, Hsinchu 30015, Taiwan; 5 Department of Ophthalmology, Far Eastern Memorial Hospital, Taipei 220216, Taiwan; 6 Institute of Statistical and Information Science, National Changhua University of Education, Changhua 50007, Taiwan; maiblian@cc.ncue.edu.tw; 7 Departament of Optometry, Da-Yeh University, Chunghua 515006, Taiwan

**Keywords:** bifocal, extended depth of focus, monofocal, spectacle, quality of vision

## Abstract

We aimed to compare the postoperative quality of vision among patients who received extended depth of focus (EDOF), bifocal, and monofocal intraocular lens (IOL) implantation. A retrospective study was conducted, and 87 patients who underwent cataract surgery were enrolled. Patients were categorized into different groups according to IOL design, with 24, 29, and 34 individuals constituting bifocal, EDOF, and monofocal groups. Preoperative and postoperative visual acuity (VA), biometry data, refractive status, contrast sensitivity (CS), higher-order aberrations (HOAs), and a quality of vision questionnaire that consisted of 11 questions were obtained 1 month postoperatively. The Kruskal–Wallis test and Pearson’s chi-square test were applied for statistical analyses. The postoperative CDVA was better in the EDOF group than in the bifocal group (*p* = 0.043), and the residual cylinder was lower in the EDOF groups than in the other two groups (both *p* < 0.05). The CS was worse in the EDOF group than in the other two groups (all *p* < 0.05), while the spherical aberration and trefoil were lower in the EDOF group than in the bifocal group (both *p* < 0.05). In terms of the quality of vision, the scores were better in the monofocal group than in the EDOF group in seven items (all *p* < 0.05), and the quality of vision in the bifocal group was better than in the EDOF group in small print reading (*p* = 0.042). In addition, the incidence of glare was lower in the monofocal group than in the other two groups (*p* < 0.001), while the spectacle dependence ratio was significantly higher in the monofocal group compared to the other two groups (*p* < 0.001). In conclusion, the general quality of vision was better in the monofocal group compared to the bifocal and EDOF groups, while the spectacle dependence ratio was significantly higher in the monofocal group than in the other two groups.

## 1. Introduction

Cataracts are the leading cause of vision impairment and blindness worldwide, causing 15,200 cases of blindness in 2020 [1]. Intraocular lens (IOL) implantation is a procedure during cataract surgery to restore postoperative visual acuity, with an average of 121,500 cases annually from 2002 to 2010 in Taiwan [2], and 7.18 percent of patients with cataracts received surgery in China [3]. Moreover, many different designs of IOLs have been applied in past decades, of which monofocal, multifocal, and extended depth of focus (EDOF) IOLs are the most popular and have been introduced to correct preoperative refractive errors or presbyopia [4,5]. Among the different types of IOLs, there are concerns about optical results and quality: EDOF IOLs have been reported to reduce spectacle dependence among patients [6], while standard monofocal IOLs usually need the assistance of glasses for reading [7].

Traditional multifocal IOLs had higher cataract symptom scores than monofocal IOLs, while VF-14, VQOL, and patient satisfaction scores were comparable between the two groups [8]. In patients with monovision management, spectacle dependence for near vision was more prevalent than in the multifocal IOL population [9]. In other studies, multifocal IOL groups exhibited better UNVA and 80 cm intermediate visual acuity (VA) than monofocal IOL groups [10,11]. However, monofocal IOL patients always exhibited better visual function questionnaire scores than multifocal IOL patients [8,10,12]. Additionally, patients with multifocal IOL implantation experienced more severe glare but a lower spectacle dependence ratio than monofocal groups [13], despite high spectacle independence and patient satisfaction in bifocal and other multifocal IOLs [14,15,16,17]. Moreover, the principle of EDOF imaging is to elongate the depth of focus from a single focal point and make it into a focused channel to avoid decreasing the optic quality caused by multiple images [18].

Comparing EDOF IOLs to bifocal IOLs, EDOF IOLs presented better quality of vision than bifocal IOLs, in addition to having similar intermediate visual restoration [19]. In another case series that compared EDOF IOLs to trifocal IOLs, the trifocal group had significantly better near VA than the EDOF group [20]. EDOF IOLs also yielded higher contrast sensitivity (CS) in a larger nonrandomized case series, whereas trifocal IOLs yielded better near VA results [21]. Nonetheless, a rare study evaluated the quality of vision indexes among bifocal IOLs, EDOF IOLs, and monofocal IOLs separately. Additionally, the evaluation of quality of vision is always via a questionnaire, which consists of a series of questions. After the completion of the questionnaire, only the total score is used without comparing each item. Because the requests of each patient regarding the visual quality may be different, a related study to discuss the quality of vision index separately should be conducted.

Consequently, our study aimed to analyze the visual performance and quality of vision of the EDOF Symfony IOL and compare it with the bifocal Restor IOL and monofocal Sensar AR40e IOL. Other parameters such as corrected-distance visual acuity (CDVA), near-corrected visual acuity (NCVA), CS, and higher-order aberrations (HOAs) were also compared among groups.

## 2. Materials and Methods

### 2.1. Patient Selection

Data from patients who underwent cataract surgery during 2018–2020 at the Taipei Nobel Eye Clinic and Universal Eye Center Clinic were used. The inclusion criteria for this study were the presence of cataracts in both eyes, age between 50 and 80 years, and CDVA of both eyes under 20/40. Phacoemulsification cataract surgery was performed on all patients. The exclusion criteria were complicated cataract; corneal opacities or irregularities; corneal astigmatism > 1.50; diopter; severe dry eye (Schirmer’s test I ≤ 5 mm); amblyopia; anisometropia; surgical complications such as posterior capsular bag rupture, vitreous loss, or IOL tilt/decentration; coexisting ocular pathologies such as glaucoma, nondilating pupil, history of intraocular surgery, laser therapy, or retinopathy; optic nerve or macular diseases; and refusal or inability to maintain follow-up. The right eyes of patients who met the inclusion criteria were selected for the analysis.

### 2.2. Surgery Details

**Figure 1:**
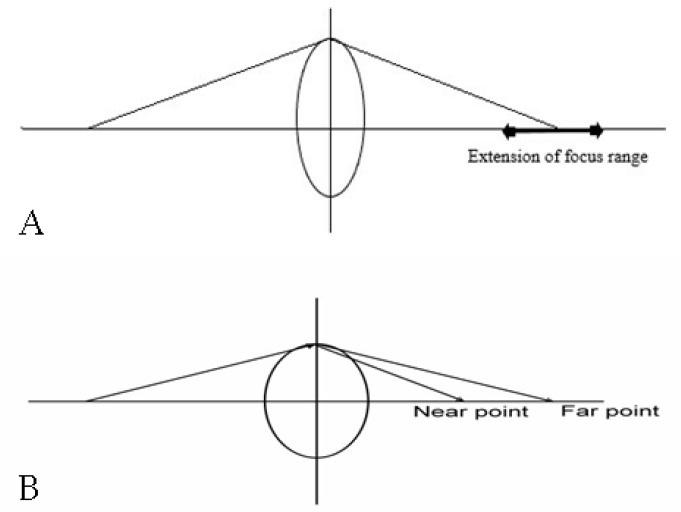
Schematic of extended depth of focus (**A**) and multifocal (**B**) intraocular lens.

Clear corneal phacoemulsification and IOL implantation were performed by two surgeons (Chao-Kai Chang and Hung-Yuan Lin) using an identical technique to minimize differences between groups. The surgical procedure involved topical anesthesia, a 3-step clear corneal incision (2.75 mm) at 180° (temporal in both eyes), a 5.0 mm continuous curvilinear capsulorhexis, phacoemulsification using the stop-and-chop technique, IOL implantation with an injector, IOL centration, and a sutureless incision. The study’s IOL models included EDOF Symfony IOL (AMO, Santa Ana, CA, USA) (Figure 1A), bifocal Restor +2.5D IOL (Alcon, Fort Worth, TX, USA) (Figure 1B), and monofocal Sensar AR40e IOL (AMO, Santa Ana, CA, USA). All surgical procedures were conducted smoothly, and all IOLs were placed in the capsular bag.

### 2.3. Ophthalmic Examinations

Patients were examined preoperatively, 1 day, 1 week, and 1 month after surgery. At each visit, tests for far- and near-uncorrected distance visual acuity (UDVA), CDVA, biomicroscopy, and applanation tonometry exams were arranged. Fundus examination was performed before surgery. Preoperatively, all patients underwent optical biometry with the IOL Master (IOLMaster 500, Carl Zeiss); calculations were performed using the SRK/T formula, and the postoperative refraction target was set at emmetropia. IOL centration was also evaluated postoperatively using retroillumination. Wavefront analysis was performed only at the 1-month postoperative visit with an AMO WaveScan Hartmann–Shack sensor (Santa Clara, CA, USA). Wavefront maps were analyzed using a 6 mm pupil diameter and a Zernike polynomial expansion up to sixth-order Zernike coefficients. Root-mean-square (RMS) errors of horizontal coma aberration (Z 3,1), spherical aberration (Z 4,0), trefoil aberration, and HOAs were assessed. Quality of vision is defined as one’s perception of vision, which could be affected by visual factors combined with psychological factors [22]. Visual acuity is tested based on the ability to recognize sharp outlines of optotypes, whereas CS is a measure of the ability to perceive slight changes in luminance that are not separated by definite borders [23]. By using a sinusoidal grating pattern, different numbers of grating periods or grating frequencies (cycles per degree (CPD)) as the horizontal axis and reciprocal of the threshold contrast as the vertical axis, the curve of a modulation transfer function (MTF) can be plotted. The MTF shifts to the left when patients report loss of visual acuity and shifts downward when patients lose CS to lower spatial frequency, resulting in vision disturbance but preservation of visual acuity [24]. CS has also been considered a measure of quality of vision in a multifocal lens study [25]. Anton et al. also used CS to assess the performance of diffractive bifocal lenses [26]. CS was also measured at the 1-month postoperative visit using the VectorVision CSV-1000 (Greenville, OH, USA) chart. All subjects were tested at a recommended distance of eight feet. The CSV-1000 consists of a series of circular achromatic sinewave patches with a 1.5-inch diameter comprising 4 rows, each corresponding to one of four spatial frequencies: 3, 6, 12, and 18 CPD. We selected 3, 6, and 12 CPD for the analysis.

### 2.4. Questionnaire Survey

The NEI-RQL-42 questionnaire was originally developed for patients with normal VA who underwent surgical correction but still had some problems related to visual function [27]. Shah et al. compared the outcomes of multifocal IOL implantation with those of monofocal IOL implantation using the NEI-RQL42 questionnaire, which assesses aspects of quality of vision, with subscales including near vision, activity limitations, dependence on correction, appearance, satisfaction with correction, clarity of vision, expectations, far vision, diurnal fluctuations, glare, symptoms, worry, and suboptimal correction. In that study, the multifocal IOL group exhibited higher spectacle independence and higher glare, as revealed by the VQOL scores [28]. For near and intermediate vision assessment, Gupta et al. developed a questionnaire for patients who received bilateral accommodating IOL implants [29]. Thereafter, Buckhurst et al. tested the questionnaire’s validity on patients who received monofocal IOL, accommodating IOL, and multifocal IOL implantation, and noted that the questionnaire had good validity and discrimination ability [30]. The near-activity visual questionnaire (NAVQ) was also used to evaluate near-vision patient satisfaction after bilateral multifocal IOL implantation [31]. The NEI-RQL-42 and NAVQ are suitable for evaluating self-reported outcomes in patients implanted with multifocal IOLs, and we used these questionnaires to evaluate the quality of vision in pseudophakic patients, evaluating far vision, diurnal fluctuation, glare and halos, spectacle dependence, near vision, and intermediate vision. In addition, subjective quality of vision was evaluated using a questionnaire adapted from a near-activity 19-item questionnaire and the NEI-RQL-42 [29,32]. Our questionnaire contains 11 questions, and the subscales include far vision, diurnal fluctuation, glare and halos, spectacle dependence, near vision, and intermediate vision. Higher item scores on the questionnaire indicated more difficulty in achieving specific visual tasks. In general, questionnaires were completed without assistance; however, at the patient’s request, explanations of the questions were provided.

### 2.5. Primary and Secondary Outcome

The primary outcome in the current study was set as the difference in each quality of vision question in the questionnaire, including the presence of glare and spectacle independence rate, among the three IOL groups. On the contrary, the secondary outcomes in our study included the postoperative VA, refractive error, CS, and HOAs. Preoperative demographics, although not regarded as outcomes, were also presented in the current study to illustrate the baseline status of the three IOL groups.

### 2.6. Sample Size

**Figure 2:**
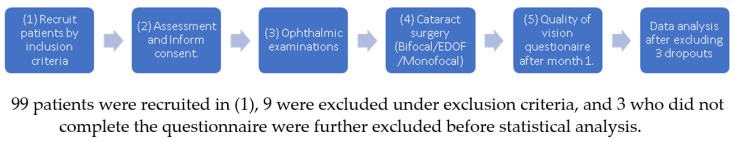
Guidelines flow diagram for quality of vision analysis.

The empirical mean difference in total score in each group from our pilot study was roughly 0.8-fold of its standard deviation, so we set the minimum sample size of 25 for each group to achieve the pre-set alpha = 0.05 and power = 0.8. Figure 2 shows the flow diagram for quality of vision analysis. Before the minimum of each group was reached, a total of 99 patients who satisfied the inclusion criteria were recruited, 9 of further examination, 9 were excluded under exclusion criteria, and 3 who did not complete the questionnaire were excluded before statistical analysis.

### 2.7. Statistical Analysis

The following four types of postsurgery measurements were used to compare the performance among EDOF, bifocal, and monofocal IOL implantation: (i) Ophthalmic examinations: UDVA, CDVA, and uncorrected near visual acuity (UNVA) (40 cm); (ii) wavefront examination: HOAs, coma, spherical aberration, trefoil, (3) contrast sensitivity 3-CPD, 6-CPD, and 12-CPD with glare off and on, respectively; (iv) quality of vision questionnaire: score of 11 questions and their mean total score. We applied the analysis of covariance (ANCOVA) to compare the above measurements among the three IOL methods, with the following covariates being adjusted: age, sex, axial length, corneal K, IOL power, and preoperative spherical equivalent (SE). The adjusted mean of the response variables for each IOL group was calculated using ANCOVA, and a pairwise comparison between IOL methods was made with EDOF as a reference. We used Rasch analysis [33] to evaluate the quality of vision questionnaire and assess whether all items measured a single underlying construct. The raw ordinal scores were then converted to interval scores and were used in the parametric statistical tests. We also used Rasch analysis to assess item hierarchy (ordering of items from least to most difficult) and person separation statistics (distinction between groups of participants based on the extent of the underlying construct) with a person separation index of 2.0. The mean-square outfit statistics of each item were set as 0.80 to 1.20. We transferred items that fit the Rasch model from ordinal data to numerical data in the range of 0 to 100. The questionnaire data were analyzed using WINSTEPS version 4.4.6 (Linacre, Winsteps.com, Chicago, IL, USA). The normality of the data samples was evaluated using the Shapiro–Wilk W test. To analyze the primary outcome measure, statistical significance was set at *p* < 0.05. Other statistical tests included the Kruskal–Wallis rank test for continuous data, Dunn’s test for post hoc estimation, and Pearson’s chi-squared test for ordinal data. Stata version 13.0 (StataCorp LP, College Station, TX, USA), was used for data analysis.

## 3. Results

Among the 87 patients, 24 received bifocal IOL implantation, 29 received EDOF IOL implantation, and 34 received monofocal IOL implantation. Table 1 presents the data on preoperative demographics and visual acuity. No significant preoperative differences in sex, corneal keratometry, or CDVA were observed. The axial length was lower in the monofocal group (23.18 ± 0.73 mm) than in the bifocal group (24.03 ± 1.46 mm) and EDOF group (24.65 ± 1.53 mm). SE was more hyperopic in the monofocal group (1.01 ± 2.20 D) than in the bifocal group (−1.05 ± 3.98 D) and EDOF group (−2.45 ± 4.69 D). Table 2 presents a comparison between the Restor bifocal IOL and Symfony EDOF IOL according to previous data [19,34].

Postoperative visual outcomes at 1 month are shown in Table 3. The mean postoperative sphere was more myopic in the EDOF group (−0.05 ± 0.50 D) but with no significant difference compared with the bifocal group (0.04 ± 0.36 D) and the monofocal group (0.04 ± 0.45 D). The postoperative cylinder in the EDOF group was higher than that in the bifocal and EDOF groups. None of the patients required further laser enhancement surgery. The UNVA in the bifocal group was better than that in the EDOF group. The CDVA in the EDOF group (0.02 ± 0.02 logMAR) was significantly better than the bifocal group (0.09 ± 0.02 logMAR).

Postoperative CS data at 1 month are also shown in Table 3. The CS at all spatial frequencies was higher in the EDOF group than in the bifocal and monofocal groups. We also compared CS in the mesopic condition with and without glare light, and a sign test of matched pairs indicated a significant decrease under glare light in 3 CPD (*p* < 0.001) and 6 CPD (*p* = 0.002) in the monofocal group and a significant decrease in 3 CPD in the bifocal group (*p* = 0.002), while in the EDOF group, the CS did not significantly decrease under glare light conditions.

Table 4 presents the item scores in our questionnaire after the Rasch transformation (scale, 0–100). To fit the unidimensional Rasch model, Item 6, “glare”, and Item 7, “spectacle dependence”, were not included in the Rasch transformation. Rasch scaling is used to convert logits to a linear scale of 0 to 100, with higher scores indicating poorer quality of vision. Table 5 presents the subjective optical quality data obtained from the questionnaire responses. For vision tasks, patients with monofocal IOL were more satisfied with the items “far vision”, “judging distances”, “getting used to the dark”, “driving at night”, and “diurnal fluctuation” than the EDOF group, and there were no significant differences between the EDOF group and the bifocal group. For near-vision tasks, patients in the bifocal group were more satisfied with the item “reading small print” than the EDOF group (*p* = 0.042). The subscale of glare for subjective optical quality is presented in Table 6. Patients in the bifocal and EDOF groups felt more glare than in the monofocal group (*p* < 0.001). The subscale of spectacle dependence for subjective optical quality is shown in Table 7. Patients in the monofocal group were more spectacle dependent for near vision compared with the bifocal and EDOF group (*p* < 0.001).

## 4. Discussion

The current study showed fair UDVA, CDVA, and UNVA for the EDOF IOL. As in Pandit’s study, the UDVA, CDVA, and UNVA were 0.02 ± 0.09, −0.05 ± 0.06, and 0.12 ± 0.09, respectively [35]. The UDVA in the EDOF group was comparable with that in the bifocal group, while the CDVA in the EDOF group was significantly better; our study result was similar to that of Pedrotti’s study: the EDOF group had comparable UDVA with the bifocal group and had significantly better CDVA than the bifocal group [7]. The mean UNVA was better in the bifocal group than in the EDOF group, and in Pedrotti’s study, the UNVA was significantly better in the EDOF group than in the bifocal group [36]. This difference may be due to different refractive target settings, that is, bilateral emmetropia in our EDOF group and mini-monovision in Pedrotti’s study.

In the present study, significantly higher mesopic CS was noted in the EDOF group at all spatial frequencies, and mesopic CS did not significantly decrease under glare light conditions in the EDOF group. Comparing CS from the EDOF IOL to the trifocal IOL, mesopic CS was also higher in the EDOF group [21]. The result in our monofocal group was different from other studies, such as Pedrotti’s study. CS in the EDOF group at all frequencies was not significantly different from the monofocal group, or as in Kohnen’s study, the CS of the EDOF IOL was less than that of the monofocal IOL [37]. The monofocal IOLs in these two studies were of aspheric design, and the CS of spherical IOLs was significantly lower than that of aspheric designs [38].

The optical quality measured by wavefront aberration in our study showed that the EDOF IOL is better than the bifocal IOL and the spherical monofocal IOL with less postoperative spherical aberration and trefoil. Our study results were consistent with those of a previous study [39], in which EDOF IOL measured less wavefront aberration compared with the bifocal IOL in a larger pupil. In Pedrotti’s study and Monaco’s study [19,20], the wavefront aberration of the EDOF IOL was comparable with bifocal or trifocal IOLs. From the studies above, we determined that the EDOF IOL could provide better or the same quality of vision compared with the bifocal or trifocal IOL.

The overall quality of vision rating for daily life activities of EDOF IOLs was similar to the bifocal IOLs in our study, although patients implanted with bifocal IOLs were more satisfied in reading small prints, which might reflect the fact that there is better UNVA in the bifocal group. Patients in the monofocal group had better satisfaction in far vision compared with the EDOF group, which might due to a higher frequency of visual disturbance in the EDOF group, consistent with Monaco’s study, in which patients in the multifocal or EDOF group had a higher incidence of visual side effects [20].

Spectacle dependence was less in the EDOF group and the bifocal group than for the monofocal group, and the result was similar to that of Monaco et al., in which higher spectacle dependence was observed in the monofocal group than in the EDOF and trifocal groups. The EDOF group had a higher proportion of spectacle dependence in the EDOF group (27.6%) than the bifocal group (12.5%) in our study, consistent with Pedrotti’s study. Patients in the EDOF group were more spectacle dependent than bifocal IOLs [19].

There were 17.2% of patients in our EDOF group not affected by halos and glare at the 1-month postoperative visit, a similar proportion of patients as in Kohnen’s study [37]. Patients in the EDOF group were more prone to perceive halos and glare compared with the monofocal group, and this trend was also noted in a recent study comparing the EDOF IOL to the monofocal IOL; the glare score measured by the NEI-RQL-42 was significantly higher in the EDOF group (78.0 ± 29.6) than in the monofocal group (66.0 ± 21.2) [36].

Our study has some limitations. First, our samples were collected retrospectively from two clinics and with relatively few case numbers, which can lead to statistical bias. Second, the different preoperative demographics, including the mean age, axial length, and spherical equivalent among the three groups could cause some errors in data interpretation, and the different filtration functions among the three IOLs may influence the results of the quality of vision. Additionally, since we used linear regression to analyze correlations between variables, nonlinear relationships may have gone undetected. Thus, a generalized additive model should be established in future studies. In addition, the effect of photic phenomena, which patients may encounter after implantation with bifocal IOLs and EDOF, and the effect of spectacle dependence, which patients may encounter after implantation with monofocal IOLs, were not included in the quality of vision questionnaire score. This limitation was due to the unidimensionality of the Rasch model. Last but not least, the quality of vision assessment (i.e., the primary outcome in the current study) is a subjective measurement. Consequently, this parameter is less reproducible and might affect the accuracy of outcome evaluation in the current study.

In conclusion, the general quality of vision is significantly better in the monofocal group than in the EDOF group and bifocal group, while the spectacle dependence rate is prominently lower in the bifocal and EDOF groups than in the monofocal group. Furthermore, the bifocal IOL performed better for reading small print than the EDOF IOL. Accordingly, the selection of IOL can be decided by asking about the patient’s favorable quality of vision items. Further large-scale study to show the different quality of vision outcomes among other newly developed multifocal IOLs and EDOF IOLs is warranted.

## Figures and Tables

**Table 1:** Preoperative demographics and visual acuity.

Parameter	Bifocal	EDOF	Monofocal	*p* Value
Mean age (SD)	64.4 (±5.9)	60.5 (±9.8)	67.4 (±6.5)	0.004 *
Patients, *n*	24	29	34	
Women	20 (83%)	17 (59%)	29 (67%)	0.243
Preop CDVA (logMAR)	0.37 (±0.29)	0.52 (±0.37)	0.45 (±0.30)	0.215
Axial length (mm)	24.03 ± 1.46	24.65 ± 1.53	23.18 ± 0.73	0.0001 *
Cornea K (D)	44.44 ± 1.98	43.83 ± 1.59	44.77 ± 1.31	0.0899
IOL power (D)	19.1 ± 4.1	18.5 ± 4.2	20.7 ± 2.0	0.0958
Preop SE	−1.05 ± 3.98	−2.45 ± 4.69	1.01 ± 2.20	0.0057 *

CDVA, corrected-distance visual acuity. * denotes significant difference.

**Table 2:** Comparison between bifocal Restor +2.5 D (AcrySof IQ SV25T0), EDOF Symfony (TECNIS ZXR00) and monofocal Sensar (AR40e).

Index	AcrySof IQ SV25T0	TECNIS ZXR00	AR40e
Design	With +2.50D and 7 diffractive steps, IOL with anterior apodized diffractive aspheric surface with a central refractive zone	Biconvex, wavefront-designed anterior aspheric surface, posterior achromatic diffractive surface, and echelette feature	Biconvex, aspheric-correcting optics at anterior and posterior surface
Filtration	UV and blue-light filtering	UV-blocking	UV-blocking
Optic material	Acrylate/methacrylate copolymer	Hydrophobic acrylic	Hydrophobic acrylic
Optic diameter	6.0 mm	6.0 mm	6.0 mm
Overall length	13.0 mm	13.0 mm	13.0 mm
Refractive index	1.55	1.47	1.47

**Table 3:** Adjusted mean of postoperative visual acuity, mesopic contrast sensitivity, and higher-order aberrations among different IOL groups, and pairwise comparison with EDOF as reference.

Parameters (Mean ± SD)	Bifocal (*n* = 24)	EDOF (*n* = 29)	Monofocal (*n* = 34)	*p* Value of Pairwise Comparison	
				B vs. E	M vs. E
VA (LogMAR)					
UCVA	0.08 ± 0.03	0.12 ± 0.03	0.07 ± 0.11	0.1897	0.0360 *
CDVA	0.09 ± 0.02	0.02 ± 0.02	0.04 ± 0.09	0.0430 *	0.2710
UNVA (40 cm)	0.17 ± 0.05	0.31 ± 0.05	NA	0.0266 *	NA
Refraction					
Sphere	0.04 ± 0.36	−0.05 ± 0.50	0.04 ± 0.45	0.0689	0.1250
Cylinder	−0.49 ± 0.40	−0.31 ± 0.38	−0.68 ± 0.47	0.0442 *	0.0005 *
Log CS					
Glare off					
3 CPD	1.36 ± 0.05	1.46 ± 0.05	1.28 ± 0.27	0.0296 *	0.0010 *
6 CPD	1.40 ± 0.05	1.61 ± 0.05	1.42 ± 0.24	0.0014 *	0.0008 *
12 CPD	1.02 ± 0.04	1.17 ± 0.05	1.00 ± 0.14	0.0055 *	0.0090 *
Glare on					
3 CPD	1.23 ± 0.04	1.50 ± 0.05	1.02 ± 0.06	0.0001 *	0.0000 *
6 CPD	1.40 ± 0.05	1.61 ± 0.05	1.31 ± 0.15	0.0012 *	0.0000 *
12 CPD	1.02 ± 0.04	1.18 ± 0.04	0.98 ± 0.12	0.0011 *	0.0005 *
HOAs/RMS (μm)					
Total HOAs	0.49 ± 0.04	0.46 ± 0.04	0.47 ± 0.17	0.1055	0.2185
Coma	0.23 ± 0.03	0.25 ± 0.03	0.26 ± 0.16	0.3100	0.3656
Spherical aberration	0.07 ± 0.03	0.01 ± 0.03	0.02 ± 0.14	0.0356 *	0.2676
Trefoil	0.44 ± 0.51	0.12 ± 0.09	0.41 ± 0.27	0.0003 *	0.0000 *

* denotes significant difference between the two groups. UCVA, uncorrected distance visual acuity; CDVA, corrected-distance visual acuity; UNVA, uncorrected near visual acuity; CPD, cycles per degree; CS, contrast sensitivity; RMS, root mean square; HOA, higher-order aberration.

**Table 4:** Item score after Rasch transformation (scale, 0–100).

Question	Item			
	1	2	3	4
Far vision	14.07	37.09	58.22	
Far vision-Judging distances	13.28	36.54	55.85	71.02
Far vision-Getting used to the dark	10.70	32.60	47.70	66.62
Far vision-Driving at night	12.38	33.70	50.96	60.28
Diurnal fluctuation	9.24	29.49	50.02	
Glare and halos	N/A			
Spectacle dependence	N/A			
Near vision-Reading small print	12.78	35.04	49.89	62.45
Near vision-For work and hobbies	16.05	34.85	46.46	72.27
Near vision-Overall satisfaction	11.72	34.14	46.58	67.69
Intermediate vision	12.63	35.80	53.35	75.25

N/A: not applicable because these 2 parameters cannot be fitted into the unidimensional Rasch model.

**Table 5:** Adjusted mean of score of 9 questions from quality of vision questionnaire and the total, and pairwise comparison with EDOF as reference.

Parameter	Bifocal (*n* = 24)	EDOF (*n* = 29)	Monofocal (*n* = 34)	*p* Value of Pairwise Comparison	
				B vs. E	M vs. E
Far vision	31.0 ± 16.4	31.2 ± 16.5	23.4 ± 13.7	0.485	0.0252 *
Far vision − Judging distances	32.0 ± 15.2	28.4 ± 17.3	21.9 ± 13.4	0.139	0.0633
Far vision − Getting used to the dark	33.4 ± 12.6	29.2 ± 16.1	20.0 ± 12.4	0.127	0.0085 *
Far vision − Driving at night	33.6 ± 13.6	34.7 ± 16.9	20.6 ± 13.0	0.469	0.0003 *
Diurnal fluctuation	30.5 ± 16.0	30.3 ± 16.2	23.0 ± 14.8	0.480	0.0355 *
Near vision Reading small print	22.6 ± 15.3	29.6 ± 16.7	22.8 ± 12.9	0.042 *	0.0483 *
Near vision For work and hobbies	22.8 ± 13.8	25.3 ± 13.9	24.9 ± 12.4	0.175	0.4805
Near vision − Overall satisfaction	28.8 ± 16.5	28.9 ± 15.4	21.8 ± 13.4	0.479	0.0348 *
Intermediate vision	27.9 ± 16.8	26.7 ± 17.4	21.5 ± 14.7	0.360	0.110
Total score	24.1 ± 10.7	24.4 ± 10.0	18.4 ± 9.6	0.438	0.003 *

* denotes significant difference between the two groups.

**Table 6:** Data obtained using the questionnaire subscales to evaluate the levels of postoperative optical quality in terms of glare and halos among groups.

Glare (Frequency) (χ^2^ Contribution)	Bifocal (*n* = 24)	EDOF (*n* = 29)	Monofocal (*n* = 34)	Total
None of the time	5 (1.1)	5 (2.3)	19 (5.2)	29 (8.6)
A little of the time	8 (0.0)	6 (1.2)	14 (0.9)	28 (2.1)
Some of the time	5 (0.1)	10 (4.1)	1 (4.4)	19 (8.6)
Most of the time	4 (0.9)	5 (1.3)	0 (3.5)	9 (5.8)
All of the time	2 (0.3)	3 (1.1)	0 (2.0)	5 (3.3)
Total	24 (2.4)	29 (9.9)	34 (15.9)	87 (28.3)
*p* value				<0.001

**Table 7:** Data obtained using the questionnaire subscales to evaluate the levels of postoperative optical quality with respect to spectacle dependence among groups.

Spectacle Dependence (Frequency) (χ^2^ Contribution)	Bifocal (*n* = 24)	EDOF (*n* = 29)	Monofocal (*n* = 34)	Total
Yes	3 (7.1)	8 (3.3)	34 (15.3)	45 (25.7)
No	21 (7.6)	21 (3.5)	0 (16.4)	42 (27.6)
Total	24 (14.8)	29 (6.8)	34 (31.7)	87 (53.3)
*p*-Value				<0.001

## Data Availability

The data can be obtained from the corresponding authors upon reasonable requests.
